# Themes in TikTok Videos Featuring Little Cigars and Cigarillos: Content Analysis

**DOI:** 10.2196/42441

**Published:** 2022-11-16

**Authors:** Julia Vassey, Scott I Donaldson, Allison Dormanesh, Jon-Patrick Allem

**Affiliations:** 1 Department of Population and Public Health Sciences Keck School of Medicine University of Southern California Los Angeles, CA United States

**Keywords:** cigarillo, little cigar, social media, TikTok, video, cigar, cigarette, smoker, smoking, tobacco, social media, content analysis, youth, young adult, adolescent, user generated content

## Abstract

**Background:**

Little cigars and cigarillos (LCCs) are popular tobacco products among youth (ie, adolescents and young adults). A variety of LCC-related promotional and user-generated content is present on social media. However, research on LCC-related posts on social media has been largely focused on platforms that are primarily text- or image-based, such as Twitter and Instagram.

**Objective:**

This study analyzed LCC-related content on TikTok, an audio and video–based platform popular among youth.

**Methods:**

Publicly available posts (N=811) that contained the LCC-related hashtags #swishersweets or #backwoods were collected on TikTok from January 2019 to May 2021. Metadata were also collected, including numbers of likes, comments, shares, and views per video. Using an inductive approach, a codebook consisting of 26 themes was developed to help summarize the underlying themes evident in the TikTok videos and corresponding captions. A pairwise co-occurrence analysis of themes was also conducted to evaluate connections among themes.

**Results:**

Among the 811 posts, the LCC presence theme (ie, a visible LCC) occurred in the most prominent number of posts (n=661, 81.5%), followed by music (n=559, 68.9%), youth (n=332, 40.9%), humor (n=263, 32.4%), LCC use (n=242, 29.8%), flavors (n=232, 28.6%), branding (n=182, 22.4%), paraphernalia (n=137, 16.9%), blunt rolling (n=94, 11.6%), and price (n=84, 10.4%). Product reviews had the highest engagement, with a median 44 (mean 2857, range 36,499) likes and median 491 (mean 15,711, range 193,590) views; followed by product comparisons, with a median 44 (mean 1920, range 36,500) likes and median 671 (mean 11,277, range 193,798) views. Promotions had the lowest engagement, with a median 4 (mean 8, range 34) likes and median 78 (mean 213, range 1131) views. The most prevalent themes co-occurring with LCC presence were (1) music (434/811, 53.5%), (2) youth (264/811, 32.6%), (3) humor (219/811, 27%), (4) flavors (214/811, 26.4%), and (5) LCC use (207/811, 25.5%).

**Conclusions:**

LCC-related marketing and user-generated content was present on TikTok, including videos showing youth discussing, displaying, or using LCCs. Such content may be in violation of TikTok’s community guidelines prohibiting the display, promotion, or posting of tobacco-related content on its platform, including the display of possession or consumption of tobacco by a minor. Further improvement in the enforcement of TikTok community guidelines and additional scrutiny from public health policy makers may be necessary for protecting youth from future exposure to tobacco-related posts. Observational and experimental studies are needed to understand the impact of exposure to LCC-related videos on attitudes and behaviors related to LCC use among youth. Finally, there may be a need for engaging antitobacco videos that appeal to youth on TikTok to counter the protobacco content on this platform.

## Introduction

Little cigars and cigarillos (LCCs) comprise over 97% of the US cigar market and are popular among youth (ie, adolescents and young adults) [1]. In 2021, LCCs were the third most-used tobacco product among US adolescents, with 5% reporting having used cigars in their lifetime [2]. The popularity of LCCs has been partly attributed to tobacco industry marketing that promotes a wide range of available flavors [3,4], a lack of minimum pack size requirements, price, and fewer sales restrictions than on cigarettes [4,5]. Research has shown that LCCs are associated with an increased risk of nicotine addiction and preventable chronic diseases, including cancer and heart disease [6,7].

A variety of content related to flavored LCCs exists on social media. Despite restrictions on marketing and sales of flavored cigar products in the United States [8] and restrictions on advertising of tobacco products on social media [9,10], platforms popular among adolescents [11,12] host direct ads from tobacco brands or promotional tobacco content from microinfluencers [13-16] (eg, models, bloggers, and brand ambassadors) who typically post on behalf of tobacco brands. Studies have demonstrated that promotional content from influencers may be perceived as more trustworthy than traditional advertising (eg, banner ads), since content created by influencers may be perceived as more authentic [17,18] than direct marketing. Exposure to tobacco-related content from such trusted sources may increase susceptibility to the use of tobacco products among social media users, including youth.

Prior research has demonstrated that LCCs are often discussed on social media in the context of music (eg, rap or hip-hop lyrics), humor, and other forms of entertainment; these are positive experiences that could normalize tobacco use among youth [5,19-22]. For example, research has shown that if adolescents observe friends, acquaintances, or influencers using tobacco products on social media and appearing to be happy and popular, they may perceive tobacco use as a behavior to emulate [23].

Research to date on LCC-related content on social media has mostly focused on text-based platforms like Twitter and image-based platforms like Instagram [5,19-22]. Missing from this literature is a comprehensive investigation of LCC-related content on emerging social media platforms, like TikTok. Only one study, to our knowledge, has analyzed promotional content for large cigars and Swisher Sweets on TikTok posted in 2016 through 2020 [16]. TikTok is one of the most popular video-sharing social-networking platforms among youth [12,24,25]. Posts on TikTok are short (typically less than a minute long) video clips often created and viewed by youth. Among TikTok users, 25% are between the ages of 10 to 19 years [24]. Due to its relatively younger user base, TikTok prohibits not only promoting but also posting any tobacco-related content or displaying the possession or consumption of tobacco by a minor [9]. However, despite these restrictions, the platform does contain promotional and user-generated tobacco-related content, including for LCCs [16,26], that could be accessible to youth. Although TikTok announced that content showing the use of tobacco products would not be eligible for recommendation by the platform’s algorithm to its users [27], the effectiveness of this policy remains unknown.

The goal of this study was to collect and characterize recent (2019-2021) posts about LCCs on TikTok. We used a detailed codebook that included 26 themes to conduct this content analysis. We documented the presence of videos featuring adolescents and young adults using LCCs and compared the prevalence of and user engagement with themes normalizing risky behavior (ie, showing LCC use as a fun experience) versus themes criticizing or warning others about the consequences of risky behavior (ie, showing negative health consequences of LCC use). Lastly, we described the presence of LCC marketing themes, including the promotion of flavors. This study provides evidence to keep the public health community abreast of an important tobacco-related issue at the intersection of social media and tobacco control.

## Methods

### Data Collection

TikTok data were collected by scraping (ie, electronically copying) publicly available posts that contained the hashtags #swishersweets or #backwoods. Similar hashtags have been used in prior research [5,19,21] and were chosen based on their high audience engagement compared to other LCC-related hashtags on TikTok. Prior to data collection, #swishersweets had 1.6 million views and #backwoods had 2.5 million views, while #blackandmild had 345,000 views.

A total of 1570 URLs were initially stored in a csv file. Metadata, which included the numbers of likes, comments, shares, and views per video, were also recorded. The study period ranged from January 2019 through May 2021. A total of 91% (1429/1570) of videos were posted in 2020 or 2021 and 9% (141/1570) in 2019.

The research team worked collaboratively to become familiar with the data and developed a codebook based on an inductive approach. The goal of this approach was to condense the raw audiovisual data into a summary format and report the underlying themes that were evident in the data. The unit of analysis was the post (ie, the TikTok video and the corresponding caption).

To establish interrater reliability, 2 coders analyzed a subsample of Backwoods-related posts (n=200), with percentage agreement ranging from 78% to 100%. Two additional coders analyzed a subsample of Swisher Sweets–related posts (n=149), with percentage agreement ranging from 81% to 99%. The senior author (JPA) served as the arbitrator and resolved disagreements. Four coders then analyzed all videos in the data set (N=1570) to document the presence or absence of each theme. If a theme was present in a post (ie, displayed or mentioned in a video, text overlay, or corresponding caption), it was coded as “1”; otherwise, it was coded as “0.” Each post could contain more than one LCC-related theme.

There were 358 inaccessible posts at the time of content analysis. To understand why such posts were inaccessible, the coders documented the apparent reason a post was no longer publicly available. These posts could have been taken down by TikTok’s content moderators, as indicated by the message “Video currently unavailable.” If the video was directly removed by TikTok, it was an indication that the platform was enforcing its community guidelines prohibiting tobacco-related content [28]. Posts could also have been made unavailable by the content creator due to changes in privacy settings, as indicated by the message “The account is private.” These posts were not included in the analytic sample. Additionally, posts coded as non–tobacco-related (n=401), such as videos with the hashtag #backwoods that showed wood paint or clothing dye, were also excluded as being irrelevant to the study, resulting in a final analytic sample of 811 posts. In this sample, the research team examined the proportion of each LCC-related theme and engagement with it, defined as the average (ie, arithmetic mean) and median numbers of likes, shares, comments, and views. Since the numbers of shares and comments were low for all categories, only the numbers of likes and views are reported.

To evaluate connections across themes (ie, the frequency with which each pair of themes was present in a single post), with the goal of uncovering the contextual complexities of a post, pairwise co-occurrence analyses were used. To visualize a co-occurrence matrix containing the counts of co-occurrences of themes, the igraph package in R (version 1.2.6; R Software Foundation) was used. Themes related to positive or negative health effects and addiction, restrictions on LCC use by adolescents, and the “other” theme were either absent or only represented by <1% of the videos and were excluded from the co-occurrence analysis.

### Ethics Approval

The University of Southern California Institutional Review Board approved all study procedures (UP-21-00135). Usernames of the content creators were not collected for this study.

## Results

The final codebook contained 26 themes (described in Table 1) that included entertainment, LCC product presence or use, presence of youth (ie, individuals who appeared younger than 30 years) in the videos, LCC product characteristics (flavors, packaging, tobacco wrap, and price), marketing and sales, user testimony, positive or negative health effects of LCC use, and addiction. Examples for each theme, represented by paraphrased quotes or descriptions of the videos, are provided in Multimedia Appendix 1.

The prevalence of the themes in TikTok videos with the #backwoods and #swishersweets hashtags are shown in Table 2. The most prominent themes in the videos (N=811) were LCC presence (ie, LCC products were shown, as opposed to only discussed; n=661, 81.5%); music (n=559, 68.9%); youth displaying, discussing, or using LCCs (n=332, 40.9%); humor (n=263, 32.4%); LCC use (n=242, 29.8%); flavors (n=232, 28.6%); branding (ie, LCC logos; n=182, 22.4%); paraphernalia (n=137, 16.9%); blunt rolling (n=94, 11.6%); and price (n=84, 10.4%). The least prevalent themes (ie, restrictions on LCC use by adolescents, other, health warnings, and risk-taking) were present in <1% of the videos for each category. Themes related to positive or negative health effects and addiction to LCCs were absent in the analytic sample (since they were present in videos that were removed by TikTok content moderators or had been made private by users at the time of content analysis).

Among themes identified in more than 1% of the videos in the sample, product reviews had the highest engagement, with a median 44 (mean 2857, range 36,499) likes and median 491 (mean 15,711, range 193,590) views; followed by product comparison, with a median 44 (mean 1920, range 36,500) likes and median 671 (mean 11,277, range 193,798) views; negative sentiment, with a median 44 (mean 983, range 36,500) likes and median 429 (mean 7545, range 193,790) views; and positive sentiment, with a median 37 (mean 485, range 4095) likes and median 563 (mean 4128, range 27,898) views. Promotions had the lowest engagement, with a median 4 (mean 8, range 34) likes and median 78 (mean 213, range 1131) views (Table 2).

**Figure 1:**
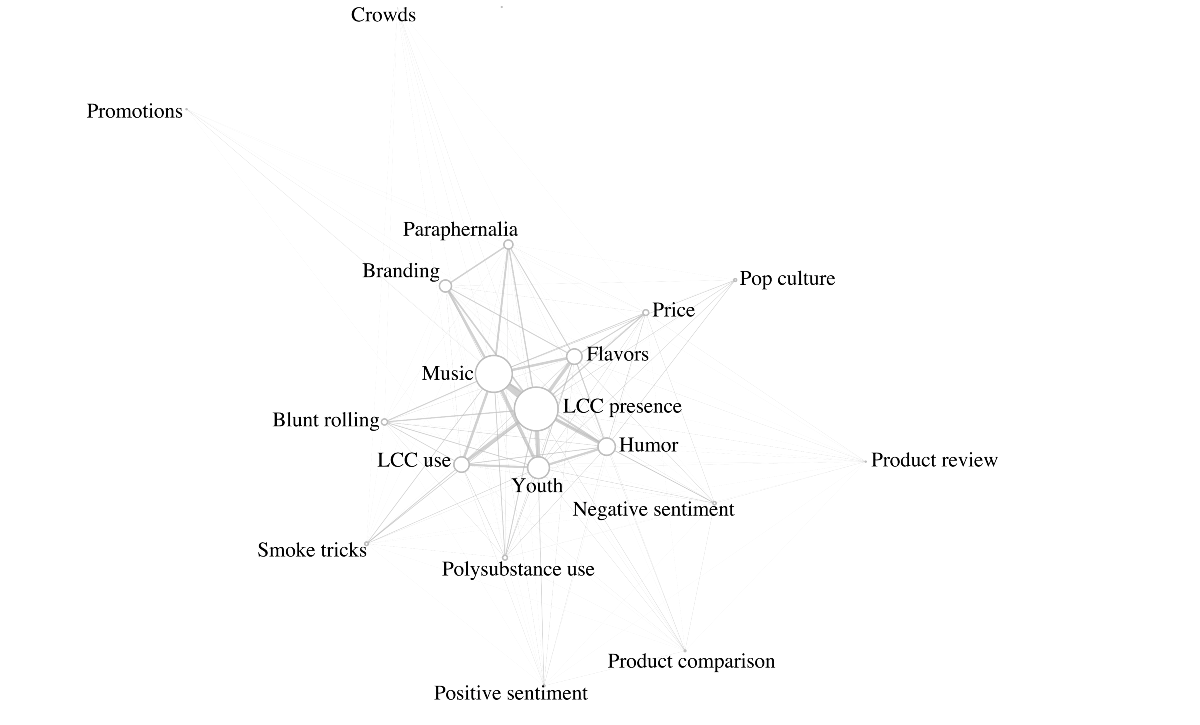
Co-occurrence of themes in little cigar or cigarillo–related videos on TikTok (N=811). The size of the circles represents the frequency with which the theme occurred. The proximity of the circles and the width of the lines represent the frequency of pairwise theme co-occurrences. LCC: little cigar or cigarillo.

As shown in Figure 1, the prevalent co-occurring themes with the LCC presence theme were music (434/811, 53.5%), youth (264/811, 32.6%), humor (219/811, 27%), flavors (214/811, 26.4%), and LCC use (207/811, 25.5%). Figure 1 in high resolution is available in Multimedia Appendix 2.

**Table 1:** LCC-related themes identified in TikTok videos collected with the hashtags #swishersweets and #backwoods. Videos unrelated to tobacco were excluded from the analytic sample.

Theme		Definition
**Entertainment**		
	Humor	Video that has a sarcastic tone, is satirical, or contains pranks, parody, or jokes (this may include memes—graphics or images that encapsulate a concept or catchphrase)
	Music	Song including a chorus or instrumentals
	Smoke tricks	Performing tricks with smoke
	Pop culture	References to rap, hip-hop, or celebrity endorsements (eg, Snoop Dogg or Cardi B)
**Product presence or use**		
	LCC^a^ presence	Visible LCC product packaging, product wrappers, or products displayed on a table, with or without product *use*; other tobacco products may be present along with LCCs (videos without the *presence* of actual products, such as those with people discussing, but not showing, LCCs or smoke clouds, were not included in this category)
	LCC use	Self-reported or visible LCC use, including puffing, hitting, rolling, and blowing smoke or an individual holding an LCC
	Blunt rolling	Blunt making (ie, the hollowing out of an LCC and refilling it with cannabis)
	Polysubstance use	Use of an LCC in combination with other substances, such as other tobacco products, alcohol, marijuana, or other illicit drugs
**Youth**		
	Youth	Presence of young people (ie, a person in the video appears younger than 30 years)
	Restrictions on LCC use by adolescents	Use by adolescents of LCCs during or after school hours on school grounds, possibly combined with disciplinary action from parents, schoolteachers, or other authority figures for using, or attempting to purchase, tobacco (before reaching the legal minimum age of 21 years)
**Product characteristics**		
	Flavors	Visible flavored LCC products, including flavor names on product packaging
	Product review	Unboxing or removing items from their packaging and showing them to viewers
	Price	Showing or discussing LCC prices, including their affordability
**Marketing and sales**		
	Branding	Visible LCC logos on merchandise, apparel, and accessories, including, but not limited to, matches, lighters, and ashtrays
	Promotions	Discussing free products, discounts, or coupons (posts in this theme may contain URLs or provide a store’s physical address or other contact information)
**User testimony**		
	Product sentiment (negative)	Expressions of dissatisfaction with LCCs or disdain for their use (this may include descriptions of the product’s quality, burning speed, or dislike of the flavors)
	Product sentiment (positive)	Expressions of approval of an LCC (this may include descriptions of the product’s quality, burning speed, or enjoyment of the flavors)
	Product comparison	Comparisons between more than one LCC product (ie, one product being better or worse than another)
**Positive or negative health effects and addiction**		
	Addiction	Discussions of addiction to nicotine because of LCC use (these posts may demonstrate a person expressing cravings for or a desire for LCCs)
	Health warnings	Expressions of concern about the health effects or adverse effects of LCC use, including lung failure, tongue discoloration, and cancer (these videos may contain scenes of a physician or medical provider offering medical advice; they do not include videos showing the nicotine warning label often seen on tobacco packages)
	Cessation	Quitting nicotine or giving up LCCs
	Risk-taking	Participating in a dangerous behavior to obtain or use an LCC (this may include cliff diving into a lake, sticking an arm into a vacuum, or inducing vomiting or other forms of self-harm)
**Other themes**		
	Paraphernalia	Visible LCC-related materials, such as matches, lighters, and ashtrays, that may or may not include LCC brand logos
	Crowds/socializing	Videos showing groups of people (more than 3)
	Other	Tobacco-related posts that do not naturally fit into the abovementioned themes
	Nontobacco	Content unrelated to tobacco
Video currently unavailable		Post removed by TikTok
The account is private		Post made private by a content creator

^a^LCC: little cigars and cigarillos.

**Table 2:** Prevalence of themes featured in TikTok videos discussing the #backwoods and #swishersweets hashtags related to little cigars and cigarillos (N=811).

Themes		Videos, n (%)	Likes, median	Likes, mean	Views, median	Views, mean
**Entertainment**						
	Music	559 (68.9)	15	257	228	2478
	Humor	263 (32.4)	20	624	322	6732
	Smoke tricks	54 (6.7)	12	694	189	3843
	Pop culture	41 (5)	20	549	247	2822
**Product presence or use**						
	LCC^a^ presence	661 (81.5)	16	217	242	2529
	LCC use	242 (29.8)	12	299	204	2365
	Blunt rolling	94 (11.6)	16	280	225	4229
	Polysubstance use	70 (8.6)	25	267	277	3438
**Youth**						
	Youth	332 (40.9)	21	483	298	4917
	Restrictions on LCC use by adolescents	4 (<1)	24	112	301	2474
**Product characteristics**						
	Flavors	232 (28.6)	18	350	225	3708
	Product review	13 (1.6)	44	2857	491	15,711
	Price	84 (10.4)	23	564	264	3994
**Marketing and sales**						
	Branding	182 (22.4)	15	953	204	6602
	Promotions	13 (1.6)	4	8	78	213
**Other themes**						
	Paraphernalia	137 (16.9)	13	1564	206	10,619
	Crowds/socializing	10 (1.2)	8	28	229	946
	Other	1 (<1)	11	11	386	386
**User testimony**						
	Product sentiment (negative)	49 (6)	44	983	429	7545
	Product sentiment (positive)	27 (3.3)	37	485	563	4128
	Product comparison	23 (2.8)	44	1920	671	11,277
**Positive or negative health effects and addiction**						
	Addiction	0 (0)	0	0	0	0
	Health warnings	1 (<1)	16	16	187	187
	Cessation	0 (0)	0	0	0	0
	Risk taking	1 (<1)	107	107	7359	7359

^a^LCC: little cigar or cigarillo.

## Discussion

### Principal Findings

We conducted a content analysis of TikTok videos featuring LCCs and found that imagery of LCCs was present in most of the analyzed videos. Many videos featuring LCCs had music and humor. Videos showing LCC use; youth displaying, discussing, or using these products; flavored LCCs; and LCC brand names and logos were also prevalent.

### Entertainment-Focused LCC Videos Featuring Youth

Entertainment-related themes (eg, music or humor) have been documented in prior research on LCC-related content on social media [5,16,19-22]. In this study, we created a codebook with nuanced categories to assess the context in which LCCs were discussed, displayed, or used by individuals who appeared to be younger than 30 years. We demonstrated that young-looking individuals in TikTok videos often displayed, discussed, or used LCCs while singing, dancing, or making jokes, potentially normalizing LCC use on social media [23]. At the same time, videos focused on health-protective behaviors, such as health warnings or cessation messages, were rarely observed. Thus, TikTok appears to be a platform that commonly shows risky health behaviors in an entertainment-focused context, exposing youth to harmful tobacco-related content [3,4].

### Flavored LCC Products

LCC flavors appeared quite frequently in the videos analyzed in this study. In April 2022, the US Food and Drug Administration proposed a product standard to prohibit all “characterizing flavors” (ie, flavors other than tobacco) in cigars to protect public health, decrease tobacco-related health disparities, and advance health equity [8]. However, flavored cigars continue to command a substantial share of the cigar market, and this study demonstrates that they are commonly depicted and advertised on social media, further exposing youth to harmful products.

### Marketing and Sales of LCC-Related Content

Among themes related to marketing and sales, only branding with LCC logos was frequently observed in this study. Videos showing promotional LCC content, including product offers with discounts, coupons, or free samples, were less commonly observed, (unlike, for example, LCC-related content on Instagram [5], where promotional posts are frequent). Promotional videos also had low user engagement, with median and mean numbers of likes and views that were among the lowest in the data set. This finding can be explained by prior research on tobacco that demonstrated that sponsored content is perceived by social media users as less authentic, and consequently less engaging, than organic user-generated content [17].

### TikTok Policy Related to Tobacco Content

TikTok is now one of the most-used platforms by adolescents and young adults, having surpassed Instagram in popularity among adolescents [12,24]. In 2021, 63% of Americans between the ages of 12 and 17 used TikTok on a weekly basis, compared to 57% for Instagram [11]. Despite TikTok’s community guidelines prohibiting the display, promotion, or posting of tobacco-related content on its platform, including videos showing the possession or consumption of tobacco by a minor [9], this study demonstrated that videos showing LCCs were present. The removal of posts that violate the platform’s community guidelines is essential for protecting youth from exposure to harmful posts, including tobacco-related posts. This is accomplished through the flagging of suspected violations by content moderators [28]. In this study, 22.8% (358/1570) of the original posts collected during the study period were removed by TikTok, which indicates that its community guidelines are enforced to a degree. Further improvement in the enforcement of community guidelines is necessary.

### Influence of LCC-Related Social Media Content on LCC Use Among Youth

Although this study found that young people were featured in LCC-related videos, little is known about the influence of LCC-related content on platforms such as TikTok or Instagram on LCC uptake among adolescents and young adults. This may be especially important for a comprehensive understanding of contributing factors to racial and ethnic disparities in health. For example, non-Hispanic Black populations have a higher rate of LCC use than non-Hispanic White populations [1-3,29,30]. While non-Hispanic Black populations are known to be disproportionately targeted by marketing material from LCC manufacturers [3,4], little research exists to demonstrate marketing strategies for tobacco products that target different racial or ethnic groups in the United States on social media. Such research could include, for example, a survey focused on assessing the effect of exposure to promotional LCC-related TikTok content on LCC use among adolescents with geographically, socioeconomically, and racially diverse backgrounds. This could contribute to a better understanding of the impact of social media marketing for LCCs on diverse youth populations in the United States.

### Limitations

Videos with only 2 hashtags (#backwoods and #swishersweets) were collected. Given the degree of user engagement with these 2 hashtags, as described in the Methods section, and the fact that Backwoods and Swisher Sweets represent 2 of the most popular LCC brands in the United States [5], we believe the decision to limit our study to these 2 hashtags was justified. However, it is possible that the sample used in this study was not generalizable to other LCC brands linked to hashtags. Additionally, this study focused on TikTok posts, and the findings may not be generalizable to other social media platforms. The posts in this study were collected from a 29-month period (24 months in 2019-2020 and 5 months in 2021) and may not extend to other time periods. In addition, we assumed that user-engagement behavior (eg, liking, viewing, commenting, and sharing posts) was constant throughout the studied period, regardless of any external factors that might have affected this behavior. Data collection relied on the public availability of posts, which prevented the collection of posts from private accounts. Many posts that were initially collected in this study were made unavailable by users or removed by TikTok administrators. This loss of data may have biased the current findings. The findings may not be generalizable to all TikTok users, since we only collected English-language videos. The coders’ assessment of the perceived age of the individuals who appeared to be younger than 30 in the videos did not allow for distinguishing between adolescents and young adults. In addition, this subjective assessment could have been biased. However, the 30-year age threshold was conservative enough to give a fairly accurate estimation of the prevalence of young-looking people featured in the videos [31]. Finally, while many videos we reviewed appeared to be user-generated, they might in fact have been sponsored and lacked the required sponsorship disclosures [13,32]. An analysis of the content creators was beyond the scope of this study.

### Conclusions

LCC-related content, including videos featuring young individuals discussing, displaying, or using LCCs, might remain on TikTok in violation of the platforms’ own community guidelines. Further improvement in the enforcement of TikTok community guidelines and additional scrutiny from public health policy makers is necessary for protecting youth from exposure to tobacco-related posts. Observational and experimental studies are needed to understand the impact of exposure to LCC-related videos on attitudes and behaviors related to LCC use among youth. Future research is also needed to assess LCC marketing strategies targeting different racial and ethnic groups on this platform. Finally, engaging, user-generated antitobacco content that appeals to youth and could be perceived by TikTok users as authentic may be needed to counter the protobacco content on TikTok. Such user-generated antitobacco content may complement the efforts of public health agencies and nonprofit organizations that create educational health campaigns to inform youth about the risks of using tobacco products [33].

## Appendix

Multimedia Appendix 1 Supplementary Table 1. Examples of themes featured in LCC-related TikTok videos (N=811).

Multimedia Appendix 2 High resolution Figure 1.

## Abbreviations

LCC: little cigars and cigarillos

## Data Availability

Data supporting the conclusions of this manuscript will be made available by the authors, without undue reservation and in compliance with the internal review board protocol, to any qualified researcher.
