# Supplementary material for: Themes in TikTok Videos Featuring Little Cigars and Cigarillos: Content Analysis
Source: J Med Internet Res. 2022 Nov 16;24(11):e42441. doi: 10.2196/42441 (PMC9713621; doi:10.2196/42441)
Supplement: Multimedia Appendix 1 [file jmir_v24i11e42441_app1.docx]

**Supplementary Table 1. Examples of themes featured in LCC-related TikTok videos (N=811).**

| **Theme** | **Example (description or paraphrased quote)** |
| --- | --- |
| **Entertainment** | |
| 1) Humor | A young person throws dozens of Swisher Sweets cigarillos on a bed and lies next to them. Caption text: “This is why they call me Miss Swisher Sweetie!” The “Swisher Sweet” song by DJ Squeeky accompanies the video. |
| 2) Music |  |
| 3) Pop culture |  |
| 4) Smoke tricks | A person inhales and exhales smoke clouds. |
| **Product presence or use** | |
| 5) LCC presence | A person shows a box of Blackwoods cigarillos and explains that they love “woods.” |
| 6) LCC use | A group of young people smoke cigarillos in a car. |
| 7) Blunt rolling | Point of view where a set of hands are removing the tobacco of a little cigar and replacing it with cannabis flower. |
| 8) Polysubstance use | A young person smokes a marijuana blunt, displays a package of Swisher Sweets, unpacks a cigarillo and smokes it, too. |
| **Youth** | |
| 9) Youth | A young person talks about the use of Backwoods LCCs and explains that not everybody knows how to use the “wood:” the art of it, rolling it, and holding it. |
| 10) Restrictions on LCC use by adolescents | A young person talks about their attempt to purchase LCC at a gas station and the subsequent refusal by the clerk to sell the product to them, because the person is under the legal tobacco purchasing age of 21. |
| **Product characteristics** | |
| 11) Flavors | A display of flavored Swisher Sweats cigarillos with narration suggesting to follow these three steps: unpack cigarillos, cut them and start smoking them. |
| 12) Product review | A person opens a Swisher Sweets pack, finds a broken cigarillo inside and expresses frustration over Swisher Sweets selling a used product. |
| **Marketing and sales** | |
| 13) Branding | A person gives a Backwoods cigarillo with Backwoods logo displayed on the packaging to a passenger in his car. |
| 14) Price | A young person shows Swisher Sweets cigarillos with the promotion 2 for 99 cents on the packaging. |
| 15) Promotions |  |
| **User testimony** | |
| 16) Product comparison | A young person displays packs of Blackwoods and Swisher Sweets cigarillos, unpacks and smokes a flavored Swisher Sweets cigarillo. The person expresses his dissatisfaction with artificial taste that leaves a bitter taste in their mouth. |
| 17) Product sentiment (negative) |  |
| 18) Product sentiment (positive) | A person comments about a positive effect of Swisher Sweets cigarillos when they smoke them after being tired. |
| **Positive or negative health effects and addiction** | |
| 19) Addiction | Absent in the analytic sample. |
| 20) Health warnings | A person talks about components of LCCs suggesting that they could be dangerous for one’s health because of infused dyes used in them to get the dark color. The person also explains that small leaves used in cigarillos do not help get all the tar and nicotine out. |
| 21) Cessation | Absent in the analytic sample. |
| 22) Risk taking | A driver and a passenger are involved in reckless driving as they steer a car wheel together after smoking a blunt. |
| **Other themes** | |
| 23) Paraphernalia | Visuals of hands unpacking an ashtray with Backwoods logo on it. |
| 24) Crowds/socializing | People ride a subway train. Video subtitle: “Fam smoking a ‘wood’ on my train.” (Smoking is not shown). |
| 25) Other | Visuals of a green liquid substance and a disposable e-cigarette device displayed on a table. |
| 26) Non-tobacco | Visuals of wood paint tagged with the hashtag #backwoods. |
