# Supplementary figures and images for: Themes in TikTok Videos Featuring Little Cigars and Cigarillos: Content Analysis
Source: J Med Internet Res. 2022 Nov 16;24(11):e42441. doi: 10.2196/42441 (PMC9713621; doi:10.2196/42441)

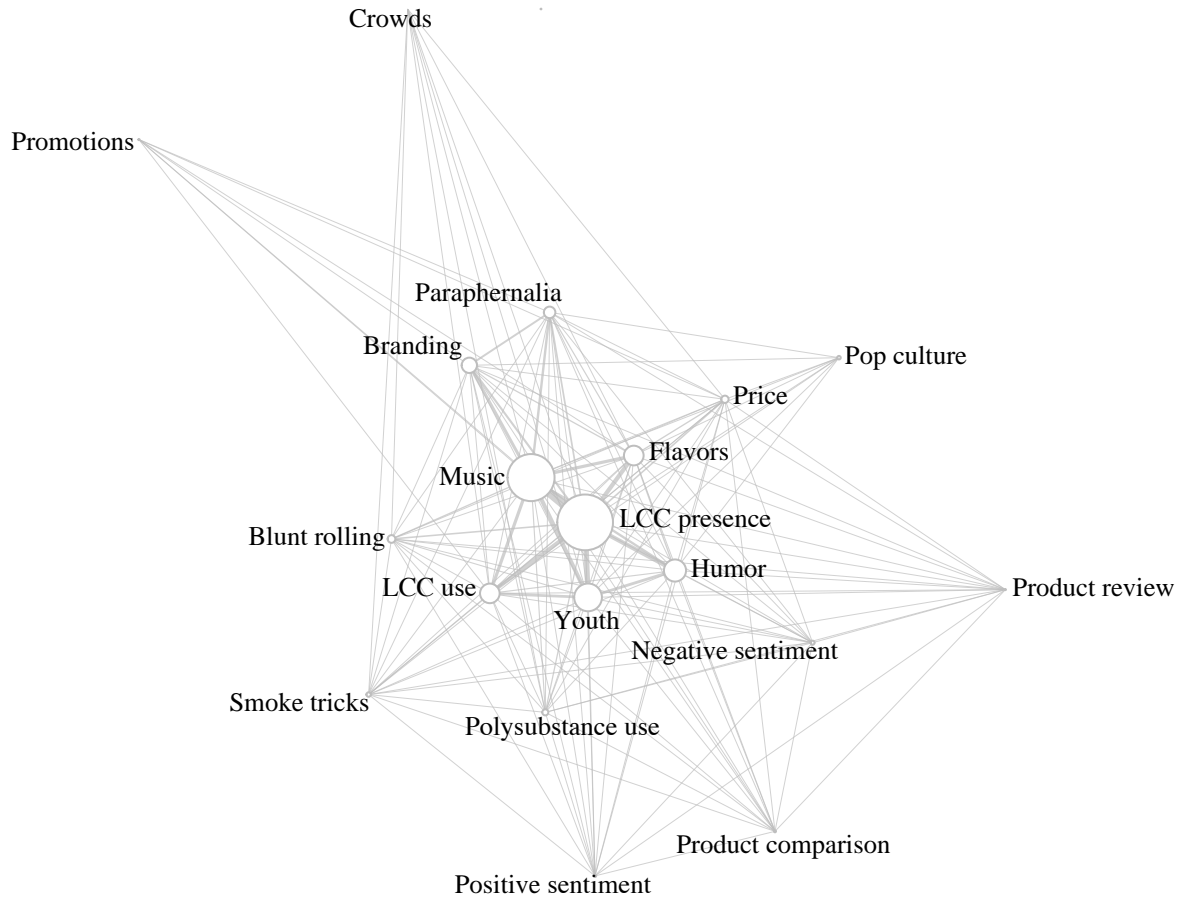

Supplement: Multimedia Appendix 2 [file jmir_v24i11e42441_app2.pdf]
